# Identification of Insulin-Mimetic Plant Extracts: From an In Vitro High-Content Screen to Blood Glucose Reduction in Live Animals

**DOI:** 10.3390/molecules26144346

**Published:** 2021-07-18

**Authors:** Verena Stadlbauer, Cathrina Neuhauser, Tobias Aumiller, Alexander Stallinger, Marcus Iken, Julian Weghuber

**Affiliations:** 1 FFoQSI GmbH-Austrian Competence Centre for Feed and Food Quality, Safety and Innovation, Technopark 1D, 3430 Tulln, Austria; 2 School of Engineering, University of Applied Sciences Upper Austria, Stelzhamerstraße 23, 4600 Wels, Austria; cathrina.neuhauser@fh-wels.at; 3 Delacon Biotechnik GmbH, Langwiesen 24, 4209 Engerwitzdorf, Austria; tobias.aumiller@delacon.com (T.A.); alexander.stallinger@delacon.com (A.S.); 4 PM International AG, 15 Wäistrooss, L-5445 Schengen, Luxembourg; marcus.iken@pm-international.de

**Keywords:** high-content screening, plant extract library, GLUT4 translocation, hen egg test, type 2 diabetes

## Abstract

Type 2 diabetes mellitus (T2DM) is linked to insulin resistance and a loss of insulin sensitivity, leading to millions of deaths worldwide each year. T2DM is caused by reduced uptake of glucose facilitated by glucose transporter 4 (GLUT4) in muscle and adipose tissue due to decreased intracellular translocation of GLUT4-containing vesicles to the plasma membrane. To treat T2DM, novel medications are required. Through a fluorescence microscopy-based high-content screen, we tested more than 600 plant extracts for their potential to induce GLUT4 translocation in the absence of insulin. The primary screen in CHO-K1 cells resulted in 30 positive hits, which were further investigated in HeLa and 3T3-L1 cells. In addition, full plasma membrane insertion was examined by immunostaining of the first extracellular loop of GLUT4. The application of appropriate inhibitors identified PI3 kinase as the most important signal transduction target relevant for GLUT4 translocation. Finally, from the most effective hits in vitro, four extracts effectively reduced blood glucose levels in chicken embryos (in ovo), indicating their applicability as antidiabetic pharmaceuticals or nutraceuticals.

## 1. Introduction

Diabetes mellitus is a metabolic disease with continually increasing numbers of patients globally. Since 1980, the number of adults suffering from diabetes has nearly quadrupled worldwide [1]. The disease leads to hyperglycemia for a prolonged period and is thought to contribute to one out of nine deaths in 20- to 79-year-old adults [2]. Two different dysfunctions cause two types of diabetes mellitus. In type 1 diabetes mellitus, insulin production in the pancreas is impaired, whereas, in type 2 diabetes mellitus (T2DM), insulin efficiency in muscle or fat tissue is reduced. With 90–95% of all diabetes mellitus patients having T2DM, it represents the most prevalent subtype [3]. Risk factors for T2DM include obesity, a change in cholesterol levels and a lack of physical exercise, which are modifiable issues, in addition to nonmodifiable factors, such as genetics or increasing age. Diagnosis of the disease often takes years because it develops gradually, and early symptoms are not severe [4]. In addition to the health effects, the economic impact of T2DM is enormous. The total cost for diabetes-related issues in 20- to 79-year-old adults was estimated to be 760 billion USD in 2019. It is expected that these expenditures will increase to 845 billion USD in 2045 [5]. Hence, the prevention and treatment of T2DM are increasing in importance.

Generally, insulin stimulates glucose uptake in myocytes and adipocytes via the translocation of glucose transporter 4 (GLUT4) from its cytosolic storage vesicles. This process takes place within minutes and is triggered by molecular signaling cascades. Insulin binds to the insulin receptor (IR), which leads to the phosphorylation of insulin receptor substrates (IRS). Activated phosphoinositide 3-kinase (PI3K) phosphatidylinositol (3,4,5)-triphosphate (PIP_3_) is enriched at the plasma membrane, which leads to activation of the Akt kinase. Activated Akt then triggers the translocation of vesicles containing GLUT4 molecules. Consequently, GLUT4 is inserted into the plasma membrane, initiating glucose uptake [6]. Additionally, there are other signaling pathways that affect GLUT4 translocation. AMP-activated protein kinase (AMPK) is a target molecule for an insulin-independent GLUT4 translocation pathway that is activated by physical exercise and energy depletion. In recent studies, it was shown that phytochemicals, such as resveratrol or carnosol, can induce GLUT4 translocation via the AMPK pathway [7,8]. Mitogen-activated protein kinase (MAPK) is a downstream molecule of AMPK, which is known to activate the MAPK pathway [9,10]. Thus, PI3K, AMPK and MAPK represent three well-known target molecules that stimulate GLUT4 translocation [11].

Natural compounds used in traditional Chinese medicine or as nutraceuticals have a long history of application for the treatment and prevention of T2DM, and several plants have been reported to possess blood glucose-reducing effects [12]. Importantly, classical pharmaceuticals exhibit stronger side effects than phytochemicals, such as hypoglycemia, osteoporosis or heart failure [13]. Therefore, additional phytochemicals that stimulate GLUT4 translocation and thereby reduce blood glucose levels need to be identified, and their mode of action needs to be studied in detail. For this purpose, different wet-lab chemistry [14,15], luminescence [16] and microscopy techniques [17,18] have been implemented, offering varying sensitivity and applicability as well as different throughput capabilities.

A highly sensitive and fast in vitro approach for the quantitation of GLUT4 translocation is based on total internal reflection fluorescence (TIRF) microscopy [19,20]. For this study, TIRF microscopy-based high-content screening was used to identify GLUT4-stimulating plant extracts from a commercial library (Plant Extract Collection Kiel in Schleswig-Holstein, PECKISH) in CHO-K1 cells [21]. Positive hits from >600 extracts were retested in additional cell lines (HeLa and 3T3-L1), and effective samples were characterized by dose–response relationships. Furthermore, the involved signal transduction pathways induced by the respective extracts were studied. Finally, the efficacy of the most promising extracts was tested in vivo using a chicken embryo model [22] to support their potential application in nutraceuticals, pharmaceuticals or food supplements.

## 2. Results

### 2.1. Identification of GLUT4 Translocation-Inducing Plant Extracts by High-Content Screening in CHO-K1 Cells

**Figure 1:**
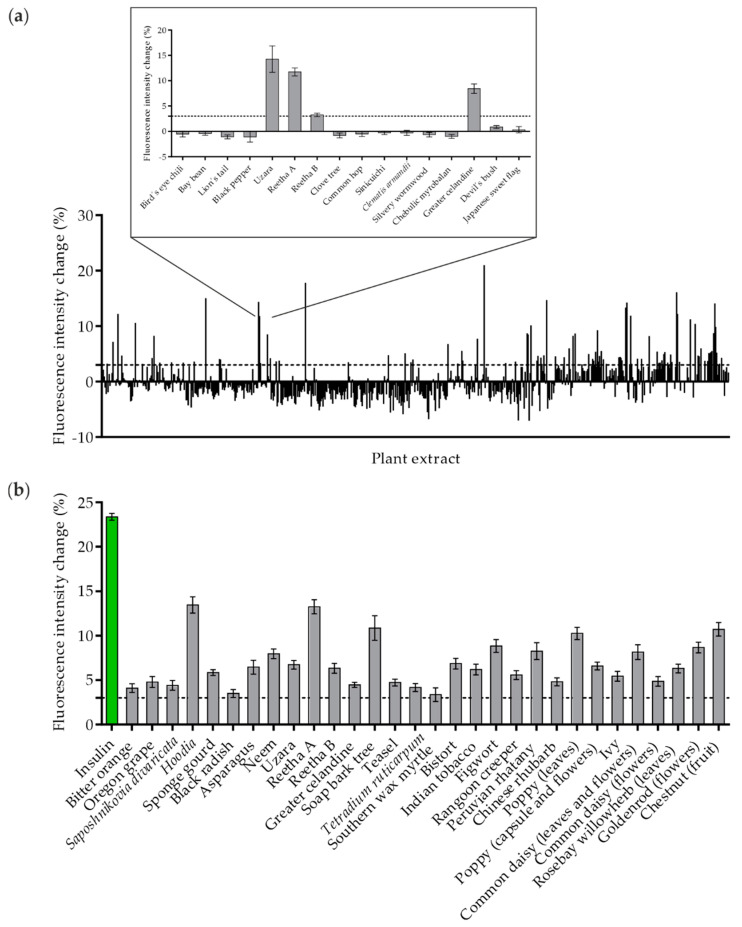
The quantification of GLUT4 translocation in a high-content screen of plant extracts in CHO-K1 hIR/GLUT4-myc-GFP cells. Cells were seeded in 96-well microtiter plates, grown overnight and then starved for 3 h in HBSS buffer. (**a**) TIRF microscopy images were taken before and after stimulation with insulin (100 nM) and 643 plant extracts (1 mg/L) for 10 min. The GLUT4-myc-GFP signal change was analyzed, and a threshold of 3% was defined for positive hits (dashed line). (**b**) Plant extracts with a positive effect, including at least three individual measurements. Data are shown as the mean ± SEM (*n* > 70).

In recent years, we have implemented and refined a TIRF microscopy approach to quantify GLUT4 translocation in live cells [17,19]. Here, we used this technique on a large scale and screened 643 aqueous plant extracts from the PECKISH library for their ability to induce GLUT4 translocation in CHO-K1 cells that are stably expressing the human insulin receptor (hIR) and a GLUT4-myc-GFP fusion protein. For analysis, starved cells were incubated with the plant extracts at a low concentration of 1 mg/L, and the increase in fluorescence intensity of the GFP signal within 10 min was determined. As indicated in Figure 1a, the primary screen resulted in 60 positive hits in a single run. A representative enlarged section shows four of 16 extracts with a positive effect of greater than a 3% signal intensity increase, which was chosen as the relevant threshold. These 60 extracts were retested at least twice, which resulted in 30 plant extracts with robust and significant efficacy (Figure 1b). Extracts prepared from *Hoodia* (genus *Hoodia*, species not defined in PECKISH), Reetha A (*Sapindus mukorossi*), soap bark tree (*Quillaja saponaria*), poppy (genus *Papaver*; species not defined in PECKISH; prepared from leaves) and chestnut (genus *Castanea*; species not defined in PECKISH; prepared from fruit) were the most effective, with an intensity increase of greater than 10%. Human insulin applied at a concentration of 100 nM was used as a positive control.

### 2.2. Validation of Positive Hits in Various In Vitro Systems

To confirm the efficacy of the 30 plant extracts identified in the first screening round, two additional cell lines stably expressing GLUT4-myc-GFP were used. On the one hand, 3T3-L1 cells represent a suitable adipocyte cell line widely applied for investigating antidiabetic compounds [23]. On the other hand, the suitability of HeLa cells has been previously described in this context [17].

**Figure 2:**
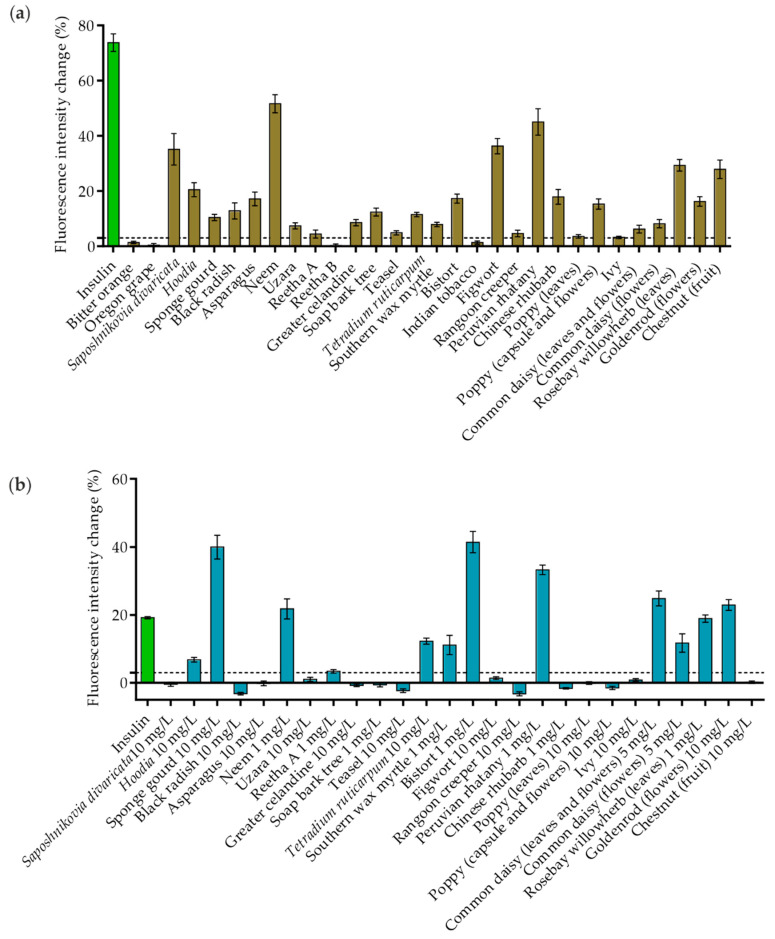
The quantification of GLUT4 translocation in 3T3-L1 and HeLa GLUT4-myc-GFP cells, including plant extracts with positive effects in the CHO-K1 screen. Cells were seeded in 96-well microtiter plates and grown overnight. (**a**) 3T3-L1 cells were starved overnight in a serum-free culture medium. TIRF microscopy images were taken before and after stimulation with insulin (100 nM) and 30 plant extracts (1 mg/L) for 10 min. The GLUT4-myc-GFP signal change was analyzed, and a threshold of 3% was defined for positive hits (dashed lines). Data are shown as the mean ± SEM (*n* > 25). (**b**) HeLa cells were starved for 3 h in HBSS buffer. TIRF microscopy images were taken before and after stimulation with insulin (100 nM) and 26 plant extracts at the indicated concentrations for 20 min. The GLUT4-myc-GFP signal change was analyzed, and a threshold of 3% was defined for positive hits (dashed lines). Data are shown as the mean ± SEM (*n* > 25).

Our studies revealed that not all of the plant extracts that stimulated GLUT4 translocation in CHO-K1 cells had the same effect in 3T3-L1 cells. As shown in Figure 2a, 26 plant extracts were reidentified as positive hits, whereas four extracts did not induce a relevant signal increase in this cell line (bitter orange (*Citrus aurantium*), Oregon grape (*Mahonia aquifolium*), Reetha B and Indian tobacco (*Lobelia inflate*)). The most effective samples were *Saposhnikovia divaricata*, *Hoodia*, neem (*Azadirachta indica*), figwort (Scrophularia nodosa), Peruvian rhatany (*Krameria lappacea*), rosebay willowherb (*Chamaenerion angustifolium*; prepared from leaves) and chestnut (genus *Castanea*; species not defined in PECKISH; prepared from fruits), with an intensity increase of greater than 20%. However, in contrast to the experiments in CHO-K1 cells, Reetha A, soap bark tree and poppy (prepared from leaves) extracts were found to stimulate GLUT4 translocation to a minor degree in 3T3-L1 cells.

In the next step, the 26 extracts with positive effects in 3T3-L1 cells were also tested in HeLa cells. These cells are known for their significant expression of the human insulin receptor, resulting in well-pronounced sensitivity to insulin, which enables their application for studying GLUT4 translocation without the need for differentiation [17]. However, for HeLa cells, the incubation time with the extracts was extended to 20 min, as the response was found to occur more slowly than in CHO-K1 or 3T3-L1 cells [17]. Additionally, the plant extract concentration was increased to 10 mg/L in the first run, as the responsiveness to insulin was lower in Hela cells. Some extracts were found to be toxic at this concentration (Reetha A, soap bark tree, common daisy (*Bellis perennis*)) or led to undesirable autofluorescence (neem, southern wax myrtle (*Myrica cerifera*), bistort (*Bistorta officinalis*), Peruvian rhatany, Chinese rhubarb (*Rheum palmatum*), rosebay willowherb). Therefore, the concentration of these extracts was reduced to 5 mg/L or 1 mg/L, respectively. As shown in Figure 2b, twelve of the 26 tested plant extracts resulted in an intensity increase in the GFP signal above the chosen threshold of 3%. Furthermore, sponge gourd (10 mg/L), neem (1 mg/L), bistort (1 mg/L), Peruvian rhatany (1 mg/L), common daisy (prepared from leaves and flowers; 5 mg/L) and goldenrod (genus *Solidago*, species not defined in PECKISH; prepared from flowers; 10 mg/L) were the most effective extracts, inducing a signal increase of greater than 20%.

### 2.3. Studying the Full Plasma Membrane Insertion of GLUT4 in HeLa Cells Treated with Plant Extracts

**Figure 3:**
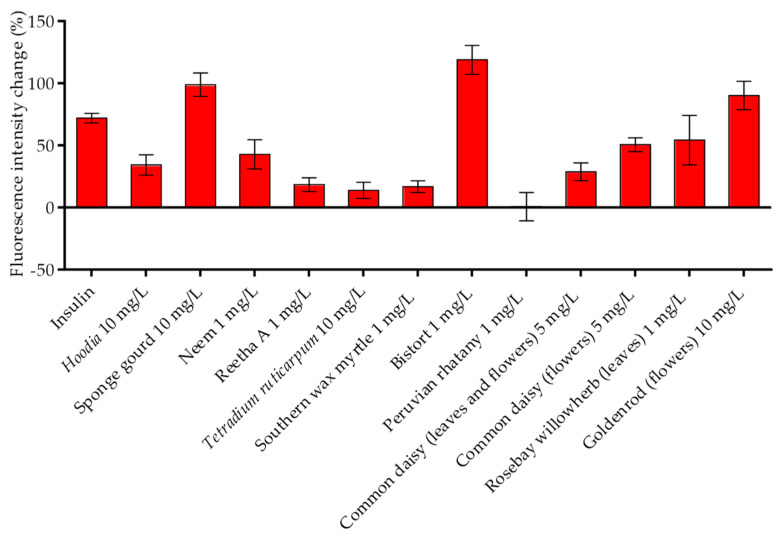
GLUT4 plasma membrane insertion induced by plant extracts in HeLa GLUT4-myc-GFP cells. Cells were seeded in 96-well microtiter plates, grown overnight and starved for 3 h in HBSS buffer. After stimulation with insulin (100 nM) or 12 plant extracts at the indicated concentrations for 20 min, the cells were fixed in paraformaldehyde and labeled using an anti-myc Alexa647 antibody. TIRF microscopy images were obtained, and the Alexa647 signal was normalized to untreated cells. Data are shown as the mean ± SEM (*n* > 50).

In our first study describing the suitability of TIRF microscopy for the quantitative analysis of GLUT4 translocation [19], we clearly demonstrated that the increase in the GFP signal in the evanescent field needs to be confirmed by effective plasma membrane insertion of GLUT4 proteins. Therefore, immunostaining using a fluorescent anti-myc antibody targeting the myc-tag in the first exofacial loop of the GLUT4 protein served as a method of choice. For this purpose, HeLa GLUT4-myc-GFP cells were starved and treated with the 12 different plant extracts that resulted in the stimulation of GLUT4 translocation in all three tested cell lines. After fixation and antibody labeling, fluorescence intensity was quantitated using TIRF microscopy compared to untreated and insulin-treated cells. As shown in Figure 3, all tested plant extracts, except Peruvian rhatany (1 mg/L), increased the fluorescence signal to various degrees, confirming GLUT4 plasma membrane insertion. Interestingly, compared to the increase in the GFP signal in live-cell experiments (Figure 2b), the fluorescence signal obtained from the myc stain was only minor for some extracts (e.g., *Tetradium ruticarpum* or southern wax myrtle). This finding suggests that some extracts induce the translocation of vesicles containing GLUT4 without a final membrane fusion step. Nevertheless, all plant extracts, with the exception of Peruvian rhatany, increased the quantity of GLUT4 in the plasma membrane.

### 2.4. Dose-Response Relationships of Effective Plant Extracts

**Figure 4:**
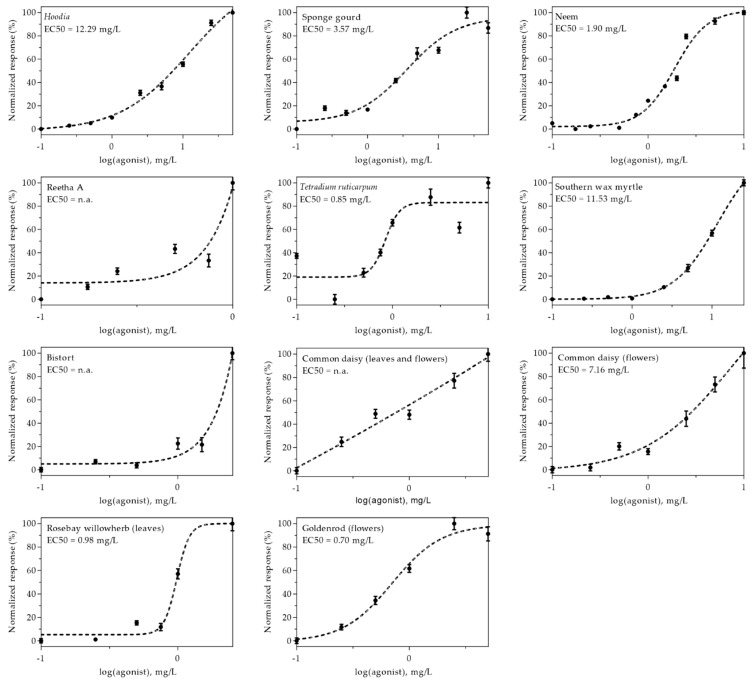
The dose–response relationship of plant extract-induced GLUT4 translocation in CHO-K1 hIR/GLUT4-myc-GFP cells. Cells were seeded in 96-well microtiter plates, grown overnight and then starved for 3 h in HBSS buffer. TIRF microscopy images were obtained before and after stimulation with various plant extract concentrations for 10 min. The GLUT4-myc-GFP signal change was analyzed, and a normalized dose–response curve was generated. The EC50 values indicate the half-maximal effective concentration (n.a. = not applicable). Data are shown as the mean ± SEM (*n* > 20).

The efficacy of eleven extracts, which were identified as the most promising insulin-mimetic substances in previous experiments, was further demonstrated by generating dose–response curves. Therefore, CHO-K1 hIR/GLUT4-myc-GFP cells were treated with the respective plant extracts in a concentration range from 0.1 to 50 mg/L. Due to toxicity and/or autofluorescence, analysis of certain extracts at higher concentrations was excluded. Normalized dose–response curves are shown in Figure 4. As indicated, some curves did not reach a plateau because higher concentrations were not applicable due to toxicity or autofluorescence. Thus, EC50 values, which indicate the half-maximum effective concentration, could not be determined for Reetha A, bistort or common daisy (prepared from leaves and flowers). However, among the other extracts, neem, rosebay willowherb (prepared from leaves) and goldenrod (prepared from flowers) were found to be the most effective. These findings also correlate in large part with the results obtained from GFP signal quantitation and anti-myc immunostaining experiments.

### 2.5. Identification of Relevant Signal Transduction Pathways Affected by Plant Extracts

**Figure 5:**
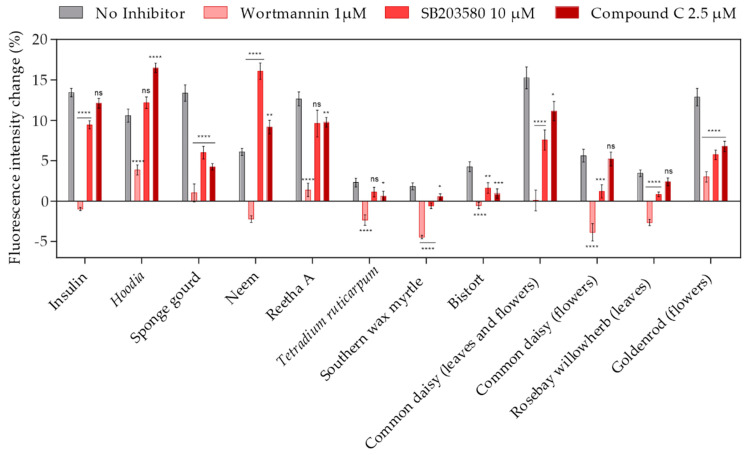
The effects of PI3K, MAPK and AMPK inhibitors on plant extract-induced GLUT4 translocation in CHO-K1 hIR/GLUT4-myc-GFP cells. Cells were seeded in 96-well microtiter plates, grown overnight and then starved for 3 h in HBSS buffer. Within the final 30 min of starvation, inhibitors of PI3K (wortmannin), MAPK (SB203580) and AMPK (Compound C) were added. TIRF microscopy images were obtained before and after stimulation with insulin (100 nM) or the plant extracts (1 mg/L) in combination with the inhibitors for 10 min, and the GLUT4-myc-GFP signal change was analyzed. Data are shown as the mean ± SEM (*n* > 30). **** p < 0.0001, *** p < 0.001, ** p < 0.01 and * p < 0.05 indicate statistically significant differences from the no inhibitor control.

Several signal transduction pathways are known to modulate GLUT4 translocation, including the AKT/IRS, AMPK and PI3 kinase pathways. [24]. To determine which of the aforementioned signaling cascades is relevant for the efficacy of the plant extracts identified in our high-content screen, specific inhibitors were used. Therefore, CHO-K1 hIR/GLUT4-myc-GFP cells were preincubated with an inhibitor for 30 min before GLUT4 translocation was quantitated by TIRF microscopy. As shown in Figure 5, the PI3K inhibitor wortmannin [25] had the strongest effect, indicating that the PI3K pathway is crucial for most of the plant extract-mediated induction of GLUT4 translocation, similar to the mode of action of insulin. Additionally, the MAPK inhibitor SB203580 [26] reduced the efficacy of some plant extracts, including sponge gourd, bistort, common daisy, rosebay willowherb (prepared from leaves) and goldenrod (prepared from flowers), but only to a minor degree. Compound C, an inhibitor of AMPK [27], also slightly reduced the efficacy of some plant extracts. Based on these results, we conclude that different signal transduction pathways are modulated by the plant extracts under study, with pronounced relevance of the PI3 kinase pathway.

### 2.6. Efficacy of Identified Insulin-Mimetic Plant Extracts in Chicken Embryos (In Ovo)

**Figure 6:**
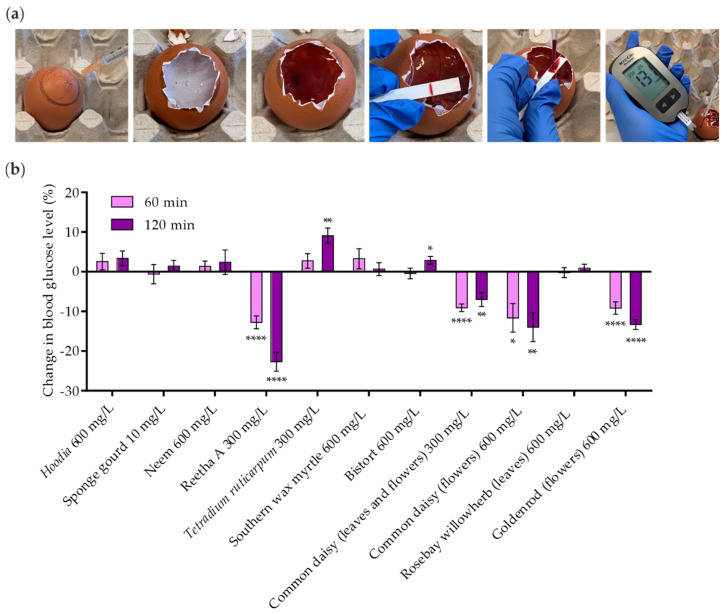
In ovo blood glucose reduction by plant extracts using Gluc-HET experiments in chicken embryos. (**a**) The air bladder was marked on the eggshell, and the chorioallantoic membrane of chicken embryos was incubated with the plant extracts for 60 and 120 min. The eggshell and the egg membrane were removed, the chorioallantoic membrane was cut, and blood was collected from a main vessel. A blood glucose meter was used for measurements. (**b**) Blood glucose levels after treatment with plant extracts at the indicated concentrations were normalized to untreated eggs and water or HBSS controls. Data are shown as the mean ± SEM (*n* > 9). **** p < 0.0001, ** p < 0.01 and * p < 0.05 indicate statistically significant differences from the water or HBSS control at the same incubation time.

In a final attempt, all extracts that induced GLUT4 translocation in vitro were investigated for their efficacy in lowering blood glucose levels in a living organism. Therefore, an assay based on chicken embryos, termed Gluc-HET [22], was used. Extracts were applied to the chorioallantoic membrane (CAM) of the chicken embryo and incubated for 60 or 120 min. As shown in Figure 6a, the eggshell and the eggshell membrane were then removed, and the glucose concentration was measured in blood collected from a major vessel. Initially, the plant extracts were applied at a concentration of 600 mg/L. In cases of toxicity, the concentration was reduced to 300 mg/L. The sponge gourd extract was toxic to chicken embryos at a concentration of 25 mg/L. Hence, this plant extract was used at 10 mg/L. Figure 6b indicates the change in blood glucose levels compared to untreated eggs, with a selected threshold of −5% for a positive evaluation of efficacy [22]. The threshold was exceeded by Reetha A (−22.7% after 120 min), common daisy (prepared from leaves and flowers; −7.0% after 120 min), common daisy (prepared from flowers; −14.0% after 120 min) and goldenrod (prepared from flowers; −13.3% after 120 min). This effect was found to be in a similar range as treatment with the insulin analogue Novo Rapid, as reported in previous studies [22]. Taken together, four extracts from three different plant species identified in our primary high-content screen lowered blood glucose levels in a living organism.

## 3. Discussion

In 2019, 463 million people were living with diabetes, which caused 4.2 million deaths worldwide [28]. Chronically elevated blood glucose levels result in micro- and macrovascular complications leading to neuropathy, diabetic foot syndrome and an increased risk of heart attack or stroke [29,30]. In the near future, the increasing number of people living with diabetes will also negatively affect the already large health and economic impacts of diabetes. Therefore, new pharmaceuticals and nutraceuticals are needed to prevent and treat diabetes. Phytochemicals represent an interesting strategy in this context, as they are associated with fewer side effects and lower costs than synthetic medication.

A straightforward strategy for identifying antidiabetic compounds is based on their efficacy in stimulating the translocation of the primary insulin-sensitive glucose transporter in adipose and muscle tissue, GLUT4 [31]. We previously introduced a microscopy-based approach that is applicable for the quantitation of GLUT4 translocation [19]. In combination with simple cell culture systems (HeLa or CHO-K1), quantification can be performed with high sensitivity and adequate throughput and is superior to other imaging technologies [17].

Here, we used this approach to identify plant extracts that induce GLUT4 translocation in the absence of insulin. In total, more than 600 samples of a commercially available extract library termed PECKISH [21] were tested at a low concentration of 1 mg/L. As a primary test system, we used CHO-K1 cells stably expressing the human insulin receptor and a GLUT4-myc-GFP fusion protein [18]. Positive hits (~5%) were subsequently retested in HeLa and 3T3-L1 cells, which represent suitable cell systems to study GLUT4 translocation [17]. The validation of efficacy also included the analysis of GLUT4 plasma membrane insertion, which is a prerequisite for glucose transport and was performed by immunostaining of the extracellular loop of the GLUT4 protein [19]. In addition, dose–response relationships were examined to further substantiate the activity of the positive hits identified in the in vitro screen. The application of all approaches resulted in the identification of 11 plant extracts with relevant insulin-mimetic activity (~2% from all screened extracts), which were tested for their effectiveness in a living system. Therefore, an improved hen egg test (Gluc-HET) was used, which has proven to be an optimal approach for the characterization of compounds with blood glucose-lowering activity in previous studies [20,22]. These experiments resulted in the identification of four plant extracts prepared from three different plant species that were effective in lowering the blood glucose concentration in living chicken embryos.

The first extract with activity in ovo was prepared from Reetha (*Sapindus mukorossi*). The fruit of Reetha, called washnut, is well known for its high saponin content and is widely used as a cleaning agent due to its surfactant properties [32]. There is currently only limited knowledge regarding its potential pharmacological effects. Some studies have demonstrated insecticidal, spermicidal, contraceptive, hepatoprotective, emetic, anti-inflammatory and anti-protozoal effects [33]. It was also shown that an extract of the stem bark of Reetha counteracts inflammatory disorders in vivo [34]. In accordance with our results, a study performed in diabetic rats indicated antihyperglycemic and antihyperlipidemic activity of washnut fruit extracts [35].

The second extract with blood glucose reducing efficacy in the Gluc-HET model was prepared from goldenrod flowers (genus *Solidago*, species not defined in PECKISH). Several pharmacological effects have been described thus far for this compound, enabling its application as a natural medicine to treat kidney disorders, urinary tract infections and prostatic diseases. Furthermore, anti-inflammatory, antiseptic and antidiabetic properties have been observed [36]. Experiments with alloxan-induced diabetic rats also demonstrated antidiabetic efficacy, i.e., a reduction in blood glucose levels and an increase in serum insulin levels [37], in good agreement with the results described in this study.

Finally, our high-content screen identified two extracts prepared from daisy flowers (*Bellis perennis*) that were effective in ovo. This potential antidiabetic activity has already been demonstrated in a preliminary proof-of-concept screen [38].

In conclusion, our TIRF microscopy-based high-content screen was successful in identifying a few plant extracts with insulin-mimetic activity and thus potential antidiabetic activity in vitro as well as in living animals. From more than 600 plant extracts that were tested in vitro, only 4 were found to be effective in ovo. However, this does not necessarily exclude the application of extracts that showed GLUT4 translocation-stimulating activity exclusively in vitro (11 extracts; ~2% positive hits) in nutraceuticals, pharmaceuticals or food supplements.

## 4. Materials and Methods

### 4.1. Reagents

pcDNA3-GLUT4-myc-GFP was a kind gift from J. E. Pessin (Albert Einstein College of Medicine, New York, NY, USA). HEPES, CaCl_2_, NaCl, KCl, MgSO_4_, KH_2_PO_4_, human insulin, wortmannin, SB203580, dorsomorphin (compound C) and paraformaldehyde (PFA) were purchased from Sigma-Aldrich (Schnelldorf, Germany). Anti-Myc/c-Myc antibody (9E10) Alexa Fluor^®^ 647 (sc-40 AF647) was obtained from Santa Cruz Biotechnology (Santa Cruz, CA, USA). Phosphate buffered saline (PBS), Hank’s balanced salt solution (HBSS), Ham’s F12 culture medium, RPMI 1640 culture medium, DMEM culture medium, penicillin/streptomycin, sodium pyruvate, G418, fetal bovine serum (FBS) and newborn calf serum (NCS) were purchased from PAN-Biotech (Aidenbach, Germany). A plant extract library containing 2300 water-soluble plant extracts (PECKISH) was obtained from Prof. Frank Döring (Christian-Albrechts University, Kiel, Germany) [21]. The plant extracts were diluted to the test concentrations in Krebs Ringer Phosphate HEPES buffer (KRPH; 20 mM HEPES, 1 mM CaCl_2_, 136 mM NaCl, 4.7 mM KCl, 1 mM MgSO_4_ and 5 mM KH_2_PO_4_).

### 4.2. Cell Culture and Transfection

CHO-K1 cells stably expressing hIR and GLUT4-myc-GFP [7] were a kind gift from Manoj K. Bhat (National Centre for Cell Science, University of Pune, Pune, India). The cells were cultured in Ham’s F12 culture medium supplemented with 100 µg/mL penicillin, 100 µg/mL streptomycin, 1% G418 and 10% fetal bovine serum (FBS). 3T3-L1 cells stably expressing GLUT4-GFP were obtained from Alan Saltiel (University of Michigan, Ann Arbor, MI, USA). The cells were cultured in DMEM high glucose culture medium supplemented with 100 μg/mL penicillin, 100 μg/mL streptomycin, 1 mM sodium pyruvate and 10% newborn calf serum (NCS). HeLa cells were obtained from ATCC (Manassas, VA, USA) and cultivated in RPMI 1640 culture medium supplemented with 100 µg/mL penicillin, 100 µg/mL streptomycin and 10% FBS. For the generation of stable clones, cells were transfected with 1–5 µg DNA at 50–70% confluence using Lipofectamine LTX Reagent with PLUS Reagent (Thermo Fisher Scientific, Vienna, Austria) according to the manufacturer’s instructions. Cells were plated into 60 mm culture dishes and grown for 48 h. The medium was removed and replaced with a medium supplemented with 400 µg/mL G418. This medium was changed every 3 days, and 15–20 days later, individual neomycin-resistant colonies were selected for propagation and analysis. For subsequent cell maintenance, 1% G418 was added to the growth medium. All cells were grown in a humidified atmosphere (~95%) at 37 °C and 5% CO_2_.

### 4.3. TIRF-Microscopy

CHO-K1 hIR/GLUT4-myc-GFP (45,000 cells/well), 3T3-L1 GLUT4-GFP (20,000 cells/well) or HeLa GLUT4-myc-GFP (45,000 cells/well) cells were seeded into 96-well imaging plates (MoBiTec, Goettingen, Germany) and grown overnight. After the cells were washed twice with HBSS, they were starved with HBSS for 3 h (CHO-K1 and HeLa) or with a serum-free culture medium overnight (3T3-L1). The cells were incubated with human insulin or plant extracts dissolved in KRPH. Images were recorded before and after substance addition at 10 min intervals. An Olympus IX-81 (Olympus, Tokyo, Japan) inverted microscope in an objective-type TIR configuration was used for imaging via an Olympus 60x NA = 1.49 Plan-Apochromat objective. Ninety-six-well plates were placed on an x-y-stage (CMR-STG-MHIX2-motorized table). Scanning of larger areas was supported by a laser-guided automated focus-hold system (ZDC2, Nikon, Tokyo, Japan). The excitation of GFP and Alexa647 was performed using 488 nm and 647 nm emission diode lasers (Toptica Photonics, Munich, Germany), respectively. After appropriate filtering, the fluorescence signal was recorded using an Orca EM-CCD camera (Hamamatsu Photonics, Herrsching, Germany).

For signaling pathway identification using the inhibitors wortmannin, SB203580 and dorsomorphin (compound C) in CHO-K1 hIR/GLUT4-myc-GFP cells, the procedure was modified as previously reported [20]. Briefly, in the starving period with HBSS buffer, the inhibitors were added for the last 30 min after 2.5 h of starvation. The PI3K inhibitor wortmannin was applied at a concentration of 1 µM, the MAPK inhibitor SB203580 at 10 µM and the AMPK inhibitor Compound C at 2.5 µM. The plant extracts were added to the cells in combination with the mentioned concentrations of the inhibitors for 10 min. TIRF microscopy imaging and data analysis were performed as described above.

### 4.4. Immunofluorescence

HeLa GLUT4-myc-GFP cells (45,000 cells/well) were grown in 96-well imaging plates overnight. The cells were starved in HBSS buffer for 3 h and incubated with insulin or plant extracts for 20 min. Afterwards, the cells were fixed in 4% precooled paraformaldehyde for 15 min on ice, followed by two washing steps with PBS and a blocking step with 5% FBS and 5% BSA in PBS for one hour at room temperature. The blocking solution was replaced with 4 μg/mL of the anti-myc Alexa647 antibody diluted in PBS for one hour. Cells were washed three times with PBS prior to the microscopy experiments.

### 4.5. Hens Egg Test (Gluc-HET)

Gluc-HET was performed as previously reported [20,22]. Briefly, fertilized eggs (Lohmann classic brown chicken) were incubated for 11 days at 38 °C with an average humidity of ~40% and turned constantly in an egg incubator (HEKA Brutgeräte, Rietberg, Germany). The eggs were checked for fertilization by candling, and the area of the air bladder was marked. In this area, the eggshell was pecked with a pointed pair of tweezers, and 300 μL of the plant extract was added with a syringe. The plant extracts were dissolved in water or HBSS. After incubation for different time intervals (60 or 120 min) at 38 °C, the eggshell above the air bladder was carefully removed. The eggshell membrane was equilibrated with PBS and then removed. The chorioallantoic membrane was carefully cut with microscissors, a suitable blood vessel was placed on a plastic pH strip, which was patted dry using filter paper before the vessel was cut, and leaking blood was collected. Blood glucose levels were determined using a blood glucose meter (Accu-Chek Performa, Roche Diabetes Care GmbH, Mannheim, Germany). For each time point, at least nine fertilized eggs were used.

### 4.6. Data Analysis

Initial imaging recordings of objective-type TIRF microscopy were supported by Olympus Xcellence RT software (version 2.1, Olympus, Tokyo, Japan). In-depth analysis for the calculation of the fluorescence intensity in individual cells and fast comparison of the fluorescent signal in numerous cells at different time intervals was performed using the Spotty framework. Each image recording from the automated large-area scan was processed as follows: all cells mapped on a single image were selected by drawing respective ROIs. Calculated mean intensity values were corrected by fluorescence background for each image (ROI drawn in an area without any cells). Spotty can be retrieved online at https://bioinformatics.fh-hagenberg.at/site/fileadmin/user_upload/img_upload/projects/spotty.html (accessed on 17 July 2021). Statistical analysis was performed using an unpaired t-test in GraphPad Prism (version 6.02, GraphPad Software Inc., San Diego, CA, USA).

## Data Availability

Not applicable.

## Sample Availability

Samples of the compounds are not available from the authors.
